# Supplementary material for: Evidence supported by Mendelian randomization: impact on inflammatory factors in knee osteoarthritis
Source: Front Med (Lausanne). 2024 May 28;11:1382836. doi: 10.3389/fmed.2024.1382836 (PMC11165061; doi:10.3389/fmed.2024.1382836)
Supplement: Supplementary file 1 [file Data_Sheet_1.ZIP › data/GCST90274784_5e6/784.leaveoneout.pdf]

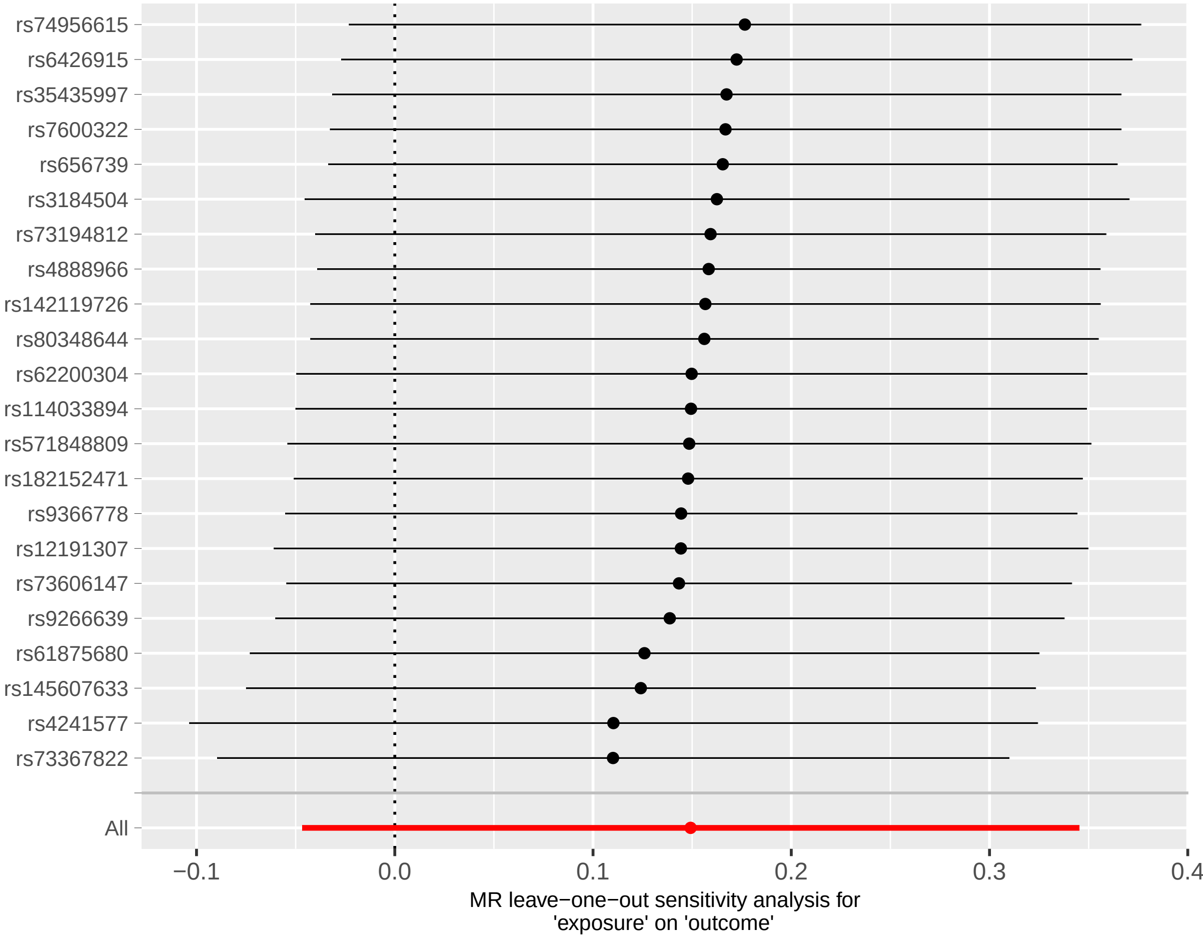

rs74956615
rs6426915
rs35435997
rs7600322
rs656739
rs3184504
rs73194812
rs4888966
rs142119726
rs80348644
rs62200304
rs114033894
rs571848809
rs182152471
rs9366778
rs12191307
rs73606147
rs9266639
rs61875680
rs145607633
rs4241577
rs73367822
All
−0.1
0.0
0.1
0.2
0.3
0.4
MR leave−one−out sensitivity analysis for
'exposure' on 'outcome'
